# Decoding the Genes Orchestrating Egg and Sperm Fusion Reactions and Their Roles in Fertility

**DOI:** 10.3390/biomedicines12122850

**Published:** 2024-12-15

**Authors:** Ranjha Khan, Muhammad Azhar, Muhammad Umair

**Affiliations:** 1 Department of Pediatrics, University of California, San Francisco, CA94143, USA; ranjha.khan@ucsf.edu; 2 The First Affiliated Hospital of USTC, Division of Life Sciences and Medicine, University of Science and Technology of China, Hefei 230001, China; axhar@ustc.edu.cn; 3 Medical Genomics Research Department, King Abdullah International Medical Research Center (KAIMRC), King Saud Bin Abdulaziz University for Health Sciences (KSAU-HS), Ministry of National Guard Health Affairs (MNGHA), Riyadh 11426, Saudi Arabia

**Keywords:** in vitro fertilization (IVF), capacitation, sperm–egg fusion, acrosome reaction

## Abstract

Mammalian fertilization is a complex and highly regulated process that has garnered significant attention, particularly with advancements in assisted reproductive technologies such as in vitro fertilization (IVF). The fusion of egg and sperm involves a sequence of molecular and cellular events, including capacitation, the acrosome reaction, adhesion, and membrane fusion. Critical genetic factors, such as IZUMO1, JUNO (also known as FOLR4), CD9, and several others, have been identified as essential mediators in sperm–egg recognition and membrane fusion. Additionally, glycoproteins such as ZP3 within the zona pellucida are crucial for sperm binding and triggering the acrosome reaction. Recent gene-editing technologies, such as CRISPR/Cas9 and conditional knockout models, have facilitated the functional annotation of genes such as SPAM1 and ADAM family members, further elucidating their roles in capacitation and adhesion. Furthermore, the integration of CRISPR-Cas9 with omics technologies, including transcriptomics, proteomics, and lipidomics, has unlocked new avenues for identifying previously unknown genetic players and pathways involved in fertilization. For instance, transcriptomics can uncover gene expression profiles during gamete maturation, while proteomics identifies key protein interactions critical for processes such as capacitation and the acrosome reaction. Lipidomics adds another dimension by revealing how membrane composition influences gamete fusion. Together, these tools enable the discovery of novel genes, pathways, and molecular mechanisms involved in fertility, providing insights that were previously unattainable. These approaches not only deepen our molecular understanding of fertility mechanisms but also hold promise for refining diagnostic tools and therapeutic interventions for infertility. This review summarizes the current molecular insights into genes orchestrating fertilization and highlights cutting-edge methodologies that propel the field toward novel discoveries. By integrating these findings, this review aims to provide valuable knowledge for clinicians, researchers, and technologists in the field of reproductive biology and assisted reproductive technologies.

## 1. Introduction

Mammalian fertilization is a biological process involving fusion between eggs and sperm and consists of multiple steps [1]. Generally, these steps involve sperm progression through the female reproductive tract, morphological and physiological changes in spermatozoa, egg–sperm adhesion and fusion, and then activation of the egg, mediated by various molecules. Morphological and physiological changes in spermatozoa involve capacitation, hyperactivation, and the acquisition of competence [2].

For many years, scientists have been investigating the molecules and mechanisms that enable egg and sperm fusion, a critical step in fertilization. Recent research has uncovered several key proteins involved in this process. One of the most important discoveries is IZUMO1, a protein found on sperm cells, which is essential for sperm–egg binding. The counterpart to IZUMO1 on the egg is JUNO, a protein located on the egg’s surface. JUNO, anchored to the egg membrane through a structure called glycosylphosphatidylinositol (GPI), plays a central role in fertilization [3].

In addition to IZUMO1 and JUNO, other proteins have been identified as significant contributors to fertilization. For example, FIMP (Fertilization Influencing Membrane Protein) is found in sperm, while CD9, a key molecule on the egg’s surface, facilitates the fusion process. Several other factors also influence fertility, including SOF1 (Sperm–Oocyte Fusion Factor 1), TMEM95 (Transmembrane Protein 95), SPACA6 (Sperm Acrosome Associated 6), SLLP1 (Sperm Lysozyme-Like Protein 1), and CD46. Additionally, the roles of chemokines such as CMTM2A and CMTM2B from the CKLF-like MARVEL transmembrane domain-containing (CMTM) protein family, as well as Equatorin (Eqtn) and sperm-specific PLCζ (phospholipase C zeta-1), are being actively studied to better understand their contributions to this intricate process [3,4].

## 2. Capacitation and the Acrosome Reaction

Artificial insemination marked a significant breakthrough in mammalian fertilization research. In the 1950s, Chang and Austin both independently introduced the concept of “capacitation”. They observed that sperm must undergo a maturation process within the female reproductive tract before penetrating the zona pellucida (ZP), a critical step in fertilization. The duration of capacitation varies across species. For example, in mice, it typically occurs within 1–2 h [5,6].

Beyond murine models, studies in other mammals, including humans and primates, have provided important insights. In primates, including humans, capacitation takes longer, often requiring several hours, reflecting species-specific differences in sperm maturation and reproductive physiology. This extended timeline allows for biochemical and structural changes, such as membrane reorganization and ion channel activation, which are essential for hyperactivation and subsequent acrosome reaction. Furthermore, experiments with hyperactivated sperm from different species, including cattle and non-human primates, have shown improved success rates in vitro fertilization, underscoring the universality of capacitation across mammals. For example, in bovine studies, the timing and conditions of capacitation have been optimized to enhance assisted reproduction technologies, while human studies continue to refine our understanding of capacitation’s role in male infertility treatment. These comparative findings highlight that while murine models have been foundational in capacitation research, exploring a broader range of species enriches our understanding and supports the development of translational applications in reproductive medicine [5,6].

### 2.1. The Acrosome Reaction and Technological Advances

Niijima and Dan [7] identified an extension of the acrosomal filament in sea urchin spermatozoa and named it the acrosome reaction [7]. In contrast to sea urchins, mammalian spermatozoa harbor acrosomal enzymes that help mediate the fusion of the acrosome membrane with the oocyte plasma membrane, referred to as the vesiculation of the acrosome. Moreover, Austin and Bishop retrieved spermatozoa from mated females of different rodents (guinea pig, golden hamster, Chinese hamster, and Libyan jird) and comprehensively studied the acrosome reaction. It was observed that the acrosomal reaction occurs prior to fertilization in mammals, and the acrosomal contents are not essential for zona pellucida penetration, as the acrosome is absent in spermatozoa that have penetrated the zona. Additionally, it is observed that the sperm head enters the egg cytoplasm, indicating that sperm penetration through the vitelline membrane occurs without actual fusion with the egg [8].

### 2.2. Zona Pellucida Penetration

The zona pellucida, a glycoprotein-rich extracellular matrix surrounding the egg, acts as a physical and biochemical barrier. Sperm penetration through the zona pellucida involves enzymatic digestion (facilitated by acrosomal enzymes such as acrosin and hyaluronidase) and mechanical force generated by sperm motility. Specific sperm-binding proteins on the zona, such as ZP3, also mediate sperm binding, further regulating fertilization specificity [9].

### 2.3. Sperm–Egg Attachment

After zona pellucida penetration, the sperm binds to the egg’s plasma membrane, also called the oolemma. This interaction involves adhesion molecules, such as Izumo1 on the sperm and its receptor Juno on the egg. The Izumo1–Juno interaction is critical for recognition and attachment, forming the first step of the fusion process. The absence of either molecule results in failed fertilization, highlighting their importance in gamete recognition. JUNO is rapidly shed from the oocyte membrane post-fertilization, a critical step in establishing the block to polyspermy. This shedding mechanism ensures that the oocyte becomes refractory to additional sperm fusion, thereby preserving the integrity of the zygote. The loss of JUNO from the oocyte surface is mediated by exocytosis of extracellular vesicles, which carry the JUNO protein away from the membrane. This process highlights the importance of JUNO not only in mediating initial sperm–egg binding but also in safeguarding against polyspermy, an essential step for successful embryonic development [4].

### 2.4. Membrane Fusion

Following attachment, the sperm and egg membranes fuse, a process mediated by fusogenic proteins such as CD9 on the egg membrane and proteins such as Fertilin-β on the sperm. This fusion facilitates the delivery of the sperm nucleus and other components, such as the centriole, into the egg cytoplasm. Membrane fusion initiates a cascade of intracellular events in the egg, including calcium oscillations, which activate the egg and trigger the resumption of the second meiotic division [10]. Fusogenic proteins also play critical roles in other biological processes, such as syncytium formation. For example, EFF-1 and AFF-1, fusogens in Caenorhabditis elegans, mediate cell fusion events during tissue morphogenesis by autonomously driving membrane merging without requiring receptor–ligand interactions. In contrast, syncytins, retroviral-derived fusogens in mammals, mediate trophoblast fusion via receptor-dependent mechanisms essential for placental development. These examples underscore the evolutionary diversity of fusogenic proteins and their specialized roles in processes ranging from fertilization to tissue and placental formation [10].

### 2.5. Egg Activation and Pronuclear Formation

Egg activation is characterized by a rise in intracellular calcium, often referred to as calcium waves. This calcium signaling orchestrates cortical granule exocytosis to prevent polyspermy and allow the completion of meiosis and the formation of the female pronucleus. Simultaneously, the sperm nucleus decondenses to form the male pronucleus, preparing for zygote formation and the first mitotic division [11].

### 2.6. Mechanisms Underlying Capacitation

It is assumed that capacitation is a process that is required to facilitate the spermatozoa’s ability to fertilize eggs by modulating the process of the acrosome reaction. Cascade changes, such as the activation of adenylate cyclase-cAMP systems and phosphorylation of sperm proteins, occur during the process of capacitation [12,13]. Mechanistic studies on capacitation demonstrated that caffeine in hamsters, heparin in bovines, and serum albumin are essential for removing cholesterol from the sperm membrane [14,15]. The success rate of IVF is severely affected by changing the amount of these factors in IVF media, indicating the involvement of these products in fertilization. In this scenario, Choi and Toyoda experimentally provided evidence that spermatozoa treated with methyl-β-cyclodextrin (MBCD) (a component that depletes cholesterol) can fertilize cumulus-free eggs and that fertilized eggs could develop under protein-free conditions [16]. These results proved that the removal of cholesterol is a key step in fertilization and that a completely protein-free, chemically defined medium can support fertilization in vitro. Hence, capacitation is the first step that enables spermatozoa to interact with eggs [16].

### 2.7. Acrosome Reaction: Key Triggers and Insights

The capacitation reaction is followed by an acrosome reaction mediated by simultaneous calcium influx in spermatozoa [17]. However, various reports portrayed that the acrosome reaction is not only triggered by calcium influx but is also controlled by many other factors, including the zona pellucida (ZP), progesterone concentration, or even spontaneously [18]. The binding of spermatozoa to O-linked oligosaccharides on ZP3 instigates the acrosome reaction, further termed the “zona-induced acrosome reaction” [19].

**Figure 1:**
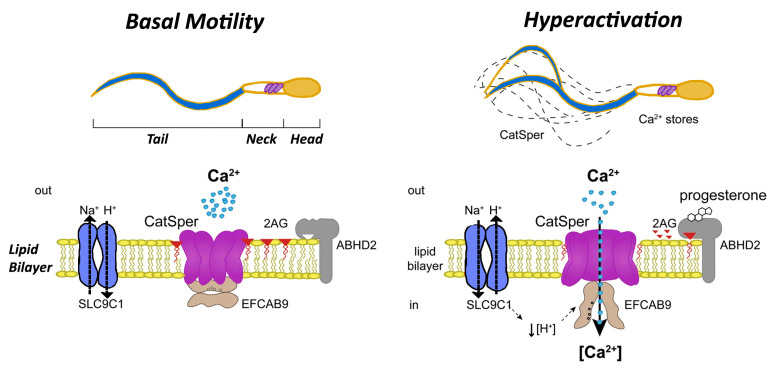
Progesterone triggers the activation of the CatSper calcium channel within an alkaline environment, causing a rapid influx of Ca^2+^ ions. This influx, combined with Ca^2+^ release from internal storage sites, raises intracellular Ca^2+^ levels. This elevated calcium concentration induces hyperactivated motility in sperm, enhancing its energy and mobility to facilitate successful fertilization.

In recent decades, the development of gene editing techniques has paved the way for studies in mutant mice, and many researchers have revisited acrosome reactions in model animals. In this context, Dean and colleagues used mutant mice and proved that the key molecule inducing sperm binding is ZP2 instead of ZP3 [20,21]. A further study revealed that sperm–egg binding relies upon the cleavage status of ZP2 [22]. On the other hand, ZP2 modification is regulated by the release of the enzyme ASTL from the cortical granules of eggs [23,24]. Thus, these studies suggested that ZP2 governs only sperm–zona interactions and that the binding of acrosome-intact spermatozoa to ZP2 (or to the zona pellucida) does not induce acrosome reactions. Interestingly, a recent study revealed that mouse spermatozoa start their acrosome reaction before contacting ZP during in vitro fertilization [25]. Furthermore, it has also been reported that mouse sperm begin to undergo acrosomal exocytosis in the upper isthmus of the oviduct [26]. Hence, the exact combination of causes that triggers the acrosome reaction is not clear, but it is believed that acrosome-reacted spermatozoa can penetrate the ZP, enter the perivitelline space, and fuse with the oolemma (Figure 1).

## 3. Major Genes Involved in the Fusion of Gametes

The fusion of gametes is fundamental in mammalian reproduction, involving numerous genes that coordinate molecular events between the sperm and egg. Key genes such as Izumo1, which is exclusively expressed on sperm surfaces, and Juno, its receptor on the oocyte, play critical roles in initial recognition and adhesion. These proteins initiate fusion, and any disruptions in Izumo1–Juno binding can lead to fertilization defects. Additionally, CD9, a tetraspanin on the oocyte membrane, aids membrane fusion and is essential for successful fertilization. Its functional partner, CD81 (Cluster of Differentiation 81), interacts with the sperm protein Izumo1, facilitating membrane merging [27]. Tetraspanins such as CD9 and CD81 play a critical role in organizing other membrane proteins into specialized microdomains, which optimize their spatial arrangement and functional interactions. This organization is vital for the efficient assembly of the molecular machinery required for sperm–egg fusion, highlighting the broader significance of tetraspanins beyond their direct fusogenic roles in fertilization [27].

**Figure 2:**
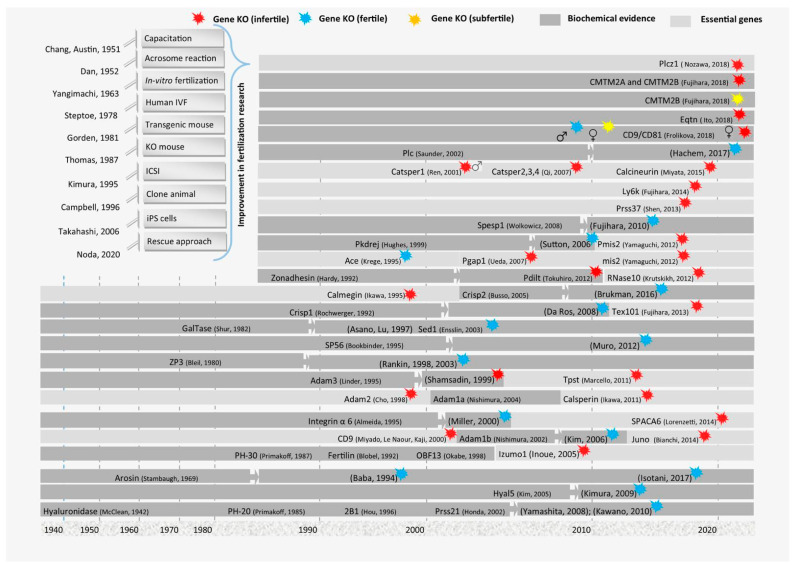
A representative outline of approaches used to discover genes that are essential for fertilization. This is the representative demonstration of genes that have been discovered through various techniques, and functional genomics analysis confirmed their role in egg–sperm fusion reactions. This figure also demonstrates the evolution of new approaches from 1950 to the present.

In recent studies, CRISPR/Cas9 (Clustered Regularly Interspaced Short Palindromic Repeats/CRISPR-associated protein 9) gene editing has pinpointed other novel factors essential for gamete fusion, such as TMEM95 in sperm and CD46 in the oocyte. Knockout (KO) experiments revealed that these genes are essential for membrane merging, as they coordinate cellular architecture changes necessary for gamete integration [28]. Calcium signaling, essential for sperm capacitation and the acrosome reaction, is regulated by genes controlling calcium channels such as CATSPER and PLCZ1. Disruptions in these pathways often lead to impaired fertilization due to a failed acrosome reaction or inability to penetrate the zona pellucida [29]. CRISPR/Cas9 and rescue techniques used to discover these genes, with a breakdown of each gene’s role and interaction, might help in understanding the mechanism in detail (Figure 2).

### 3.1. IZUMO1 and Its Associated Proteins

IZUMO1 is a stable monomeric protein with a boomerang shape. It has a secondary structure constituting α helices and β sheets. It belongs to the immunoglobulin (Ig) superfamily. The overall structure is composed of two domains: rod-shaped N-terminal residues and C-terminal IgG-like residues. It is expressed in the testes and is localized in the inner and outer acrosomal membranes of acrosome-intact spermatozoa. Once the acrosomal reaction activates, IZUMO1 is translocated to the sperm plasma membrane [2].

IZUMO1 is an essential factor in sperm and is also the main fusion factor that allows sperm to fuse with eggs. However, IZUMO1 is devoid of any type of fusion domain. Hence, it is predicted that it may interact with some associated proteins that aid in its fusion process to complete fertilization [30]. Studies have revealed that IZUMO1 can bind with oocytes but does not induce fusion, which shows that the role of IZUMO1 is as a membrane protein without fusion capability. Furthermore, its N-terminal domain forms dimers, which means that IZUMO1 can organize and stabilize the multiprotein complex that is indispensable for the function of the fusion machinery [31,32]. Another study revealed a protein, ACE3 (angiotensin-converting enzyme 3), located at the acrosomal cap of the sperm that interacts with IZUMO1 and participates in the process of fertilization. However, it was identified that ACE3 disappears after the acrosomal reaction, and its deficiency (in vivo and in vitro) did not cause infertility [33].

Recently, to check the effect of IZUMO1 on fertilization in mammals, mice were selected as a model organism, and Izumo1-deficient mice were generated by homologous recombination. These mutant mice were healthy, showing no clear symptoms of developmental abnormalities. Despite normal mating behavior, mutant mice became sterile, although they ejaculated to form vaginal plugs normally. Furthermore, the penetration of sperm in the zona pellucida was normal, but the fusion of eggs and sperm did not occur, which resulted in the accumulation of sperm in the perivitelline space of eggs [32]. The sperm were stained with an MN9 (EQUATORIN, also known as MN9 antigen, is a key marker of fertilization) monoclonal antibody, and mutant mouse sperm were clearly stained with MN9 [34]. The results showed that the sperm underwent an acrosomal reaction but failed to fuse with the eggs. Moreover, to examine whether the defect of the mutant sperm was limited to its fusion ability with the eggs or whether it extends to later stages of development, the mutant sperm was directly injected into the cytoplasm of wild-type eggs, and the later developmental stages were observed. Sperm-injected eggs were activated successfully, and embryo implantation was normal at a normal ratio [30]. Altogether, these studies revealed the essential role of IZUMO1 in sperm and egg fusion reactions during the mammalian fertilization process.

JUNO is a GPI-anchored protein and is present on the plasma membrane of oocytes. The membrane releases JUNO and then localizes itself within the perivitelline space vesicles. IZUMO1 and JUNO form a 1:1 complex [4]. The expression pattern of JUNO matches that of IZUMO1, binding with ovulated eggs. Furthermore, KO experiments showed that female mice that lacked JUNO were infertile, whereas male mice were unaffected in both the in vivo and in vitro experiments. Despite the normal zona pellucida transition, the eggs of Juno KO female mice were unable to fuse with wild-type sperm. Through electron microscopy, it was observed that the JUNO signals disappeared in the pro-nuclear stage of the early embryo. It has been assumed that this rapid disappearance of protein may be helpful in the prevention of polyspermy in mammals [35]. To directly interact with the IZUMO1 protein in solution, monomers of JUNO form clusters with each other. CD9 may enable this specific topology in the membrane of the eggs (Table 1).

**Figure 3:**
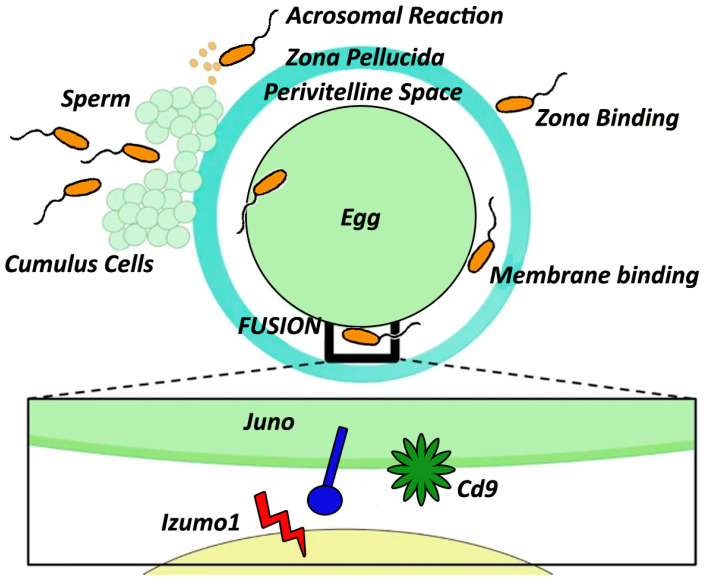
Fertilization in mammals is a complex, multi-step process. It begins with sperm interacting with cumulus cells, followed by binding to the egg’s extracellular matrix (zona pellucida) and penetrating it. Once the sperm has reached the egg cell membrane, it binds and fuses with it, triggering polyspermy-blocking mechanisms to ensure successful fertilization. Crucial to this fusion process are three core molecules: Izumo1 on the sperm, Juno on the egg, and Cd9, which work in tandem to facilitate and regulate the fusion of sperm and egg cells.

To date, several other genes have been reported that play key roles in sperm–egg interactions and assist in the addition of sperm migration in the oviduct. Thus, to generalize the concept of the complex process of the fusion reaction, we proposed a model showing the fusion reaction and sperm migration in the oviduct, along with the responsible genes (Figure 3).

### 3.2. Role of CD9 and CD81 in Mediating the Sperm–Egg Interaction

CD9 and CD81 are ubiquitous proteins belonging to the tetraspanin family [31,32]. Their ability to form tetraspanin webs and networks makes them molecular facilitators in cell activation, transduction, proliferation, the motility of somatic cells, and cellular development [36,37,38,39].

In somatic cells, CD9 and CD81 form associations with integrins. Similar associations were expected in the sperm because research revealed that the sperm head is composed of integrins [40,41]. To investigate the mutual position of CD9 and CD81 on the mouse sperm head, freshly released epididymal, capacitated, and acrosome-reacted sperm were examined, and it was confirmed that CD81 is expressed and covers the apical acrosome, whereas CD9 is expressed on the acrosomal membrane in mouse epididymal sperm [42]. It was observed that the position and localization of CD9 and CD81 remained unaffected during the capacitation process, but relocation of both proteins occurred during the acrosomal reaction. In epididymal sperm, no colocalization of CD9 and CD81 was recorded. After the acrosomal reaction, mutual colocalization was detected in the sperm head [43].

Mutual localization of CD9 and CD81 was also observed in human sperm heads. In the ejaculation of human sperm, the apical acrosomal region is composed of CD81 and CD9. In the post-acrosomal region, CD9 was not abundant. Moreover, the labeling patterns of both proteins were also different from each other. For example, a very specific and nonhomogeneous CD81 pattern in the form of dots was observed. However, CD9 showed an evenly distributed pattern. After the acrosomal reaction, CD81 disappeared from the apical region of the acrosome cap. In addition, ejaculated human sperm showed a higher degree of colocalization, while in acrosome-reacted sperm, no colocalization was observed [44]. However, only Cd81 KO female mice were sub-fertile, whereas male KO mice were completely fertile. On the other hand, Cd81 and Cd9 double-KO female mice were completely infertile, indicating that the synergistic effect of both proteins is essential for the fusion process [45].

In another study, CD9 was observed to be released by unfertilized ova in its membrane vesicle form [46]. These vesicles are secreted into the perivitelline space and then transferred to the membrane of the sperm head, hence conferring fusion competence to sperm. However, this experiment could not be reproduced in independent laboratories and yet caused little controversy [10]. Thus, to investigate the in vivo function of CD9, Cd9 KO mice were generated. Both male and female KO mice displayed normal growth and were healthy. Surprisingly, only female mice showed severely reduced fertility, while male mice were completely fertile [47]. The defects that were observed in KO female mice were rare and uncommon because wild-type sperm bind to the oolemma after penetrating the zona pellucida, although further fusion failed [48,49]. Another study revealed that CD9-null eggs showed a reduced capacity for strong sperm adhesion [50,51]. Thus, the exact role of this protein might only be in organizing the multiprotein complex and the morphology of the membrane needed for fusion.

CD9 and CD81, members of the tetraspanin family, play critical roles in sperm–egg fusion by organizing membrane microdomains and facilitating protein clustering. CD9, abundantly expressed on the oocyte plasma membrane, is essential for successful sperm–egg fusion, as demonstrated by infertility in CD9 knockout mice despite normal sperm binding and zona pellucida penetration. CD81 appears to partially compensate for CD9 loss, but its exact role remains less defined. While CD9 is thought to stabilize the IZUMO1–JUNO complex and influence membrane curvature, the precise mechanisms by which it facilitates fusion are still unclear. Questions remain regarding its direct or indirect interactions with fusion machinery, the redundancy between CD9 and CD81, and how these tetraspanins coordinate with other proteins such as SOF1 and SPACA6. Furthermore, species-specific differences and the potential therapeutic applications of targeting these tetraspanins in fertility treatments or contraceptives highlight critical areas for future research. Addressing these gaps will deepen our understanding of the molecular basis of fertilization [43,52,53].

### 3.3. EQUATORIN (EQTN) Is Necessary for Gamete Adhesion

Egg adhesion to fusion is mediated by various proteins in the sperm and egg [54,55]. Another important candidate is the type 1 acrosomal membrane protein EQUATORIN (Eqtn), which is likely to be involved in the gamete adhesion and fusion process [56,57]. Eqtn is a 40–50 kDa N, O-sialoglycoprotein preferentially integrated into the developing spermatid acrosome during spermatogenesis. It is composed of a single transmembrane domain with a C-terminus in the cytoplasm and an N-terminus in the acrosome lumen [34]. However, in acrosome-reacted sperm, the molecular size of EQUATORIN is reduced to approximately 35 kDa [58]. Therefore, the N- and C-terminal domains may be cleaved in sperms in a time-dependent manner during fertilization by a still unknown mechanism. Moreover, authentic equatorin orthologues are conserved across mammalian species, including humans.

Equatorin-deficient mice were reported to be sub-fertile due to sperm dysfunction [59,60]. Another study on Eqt^−/−^ mice further explored the role of Eqtn in the fertilization process. EQTN-KO males were normal with no apparent anomalies in terms of sexual behavior, features of reproductive organs, or spermatogenesis. However, it was observed that the average number and body weight of fetuses at 17 days post-plug formation were reduced significantly in Eqt^−/−^ males. This decrease in body weight implies the additional role of EQUATORIN in embryonic development, as some amount of EQUATORIN is incorporated in eggs from the side of the inner acrosomal membrane by an unknown mechanism. Equatorin-deficient sperm exhibited normal motility, phenotype, and acrosome reaction patterns and penetration to the zona pellucida. Sperm nuclei were able to reach the perivitelline space (PVS). Time-lapse imaging showed the vigorous movement of Eqt^−/−^ sperm in the PVS, suggesting a defect in sperm–egg adhesion. This notion was further supported by an in vitro gamete binding assay using activated sperm and zona-free oocytes that showed a significant reduction in the number of Eqt^−/−^ sperm attached to oocytes [60,61].

Another important EQUATORIN facilitator protein, i.e., SPESP1, showed aberrant staining intensity and distributions in the acrosome starting from an early-stage acrosome reaction. The cumulative effect of EQUATORIN and SPESP1 on fertility was further investigated by creating Eqtn/Spesp1^−/−^ double-KO mice. Their sperms were also able to reach and accumulate in the PVS but were unable to enter the egg cytoplasm. Furthermore, an abnormal granular IZUMO1 immunostaining pattern in the Eqtn/Spesp1^−/−^ sperm head was observed after the acrosome reaction [61]. These results further strengthen the idea that the reduction in fertility is due to sperm–egg adhesion failure.

### 3.4. FIMP Is Indispensable for Fertilization

A recent study demonstrated a testis-specific gene, 4930451I11Rik, which encodes two isoforms; a secreted form and a transmembrane form. The CRISPR/Cas9-mediated deletion of its transmembrane and the transgenic rescue approach revealed that only the transmembrane isoform plays a critical role in the fertility of mice. Therefore, this gene was renamed fertilization influencing membrane proteins (FIMPs) [62].

The fecundity of mouse spermatozoa was highly dependent on the 493041I11Rik gene, as the fusion of 493041I11Rik KO sperm with eggs was impaired. Unexpectedly, IZUMO1 was still intact in 493041I11Rik KO mice, indicating that IZUMO1 is not the sole factor necessary for the fusion process. Hence, using CRISPR/Cas9, a new gene was utilized, whose KO in mice led to the production of sperm lacking the ability for egg fusion despite intact IZUMO1.

### 3.5. SOF1, TMEM95, and SPACA6 Are Required for Sperm–Oocyte Interactions

SOF1 was identified as the protein singlet in TGCs (testicular germ cells) and as a doublet in acrosome-intact spermatozoa. It undergoes post-translational modifications after the maturation of sperm. After the acrosomal reaction occurred, the upper band of SOF1 remained intact, whereas the lower band disappeared. The upper intact band of SOF1 functions during the fusion of gametes, whereas the lower band, which disappears during the acrosomal reaction, is released from the surface of the sperm during sperm exocytosis so that other fusion factors may be activated or unmasked. Moreover, there is still a need to further characterize SOF1 to determine its more detailed functions [1].

Similarly, TMEM95 was identified as a type-1 single-pass transmembrane protein that carries a signal peptide at the N-terminus and a transmembrane helix at the C-terminal region [1]. A study revealed that a nonsense mutation in TMEM95 leads to truncation of the C-terminus, causing idiopathic subfertility in bulls [63]. Bovine spermatozoa with mutant TMEM95 were able to perform acrosomal reactions but showed a loss of fusing ability with the oocyte plasma membrane. As a result, the transmembrane domain may be vital for the innate functioning of TMEM95 in fertilization (sperm–oocyte fusion mechanism). Moreover, TMEM95 disappears in bovine spermatozoa related to acrosomes [64]. It may not function as a fusion protein but may disguise the real fusion agents before the acrosome reaction takes place.

SPACA6 was reported years ago but did not gain much attention from researchers in this field; therefore, the mechanism of this protein that is related to fusion remains unknown [65]. SPACA6 and IZUMO1 hardly share homology in their peptide sequences, whereas similarities have been identified between domains. Therefore, it is speculated that the function and behavior of SPACA6 and IZUMO1 during the fertilization process are similar. Through Tg (reporter mouse) mice, it was observed that before the acrosomal reaction, SPACA6 was intact and present at the sperm head, whereas after the acrosomal reaction, it spread out only to the equatorial segment. This finding was helpful in explaining why membrane fusion usually takes place at the equatorial segment of the spermatozoa [58].

Using tandem immunoblot assays, no interactions were observed between IZUM01 and SPACA6. In the HEKT293T (Human Embryonic Kidney 293T) cell expression system, interactions were observed. Such interactions might have resulted after the translocation of IZUMO1 or after IZUMO1 binds to JUNO [1]. Furthermore, it might be possible that SOF1, TMEM95, SPACA6, and FIMP lack any counterparts on the plasma membrane of oocytes or might have weak interactions between ligands and receptors. Hence, the HEK293T cell system expressing all five proteins failed to show any improved adhesion with zona pellucida-free oocytes. Therefore, it is said that these five proteins identified thus far are not enough for fusion to take place, and there is a dire need for future investigations to fully understand the fusion process [1]. Moreover, the overexpression of SOF1, TEME95, and SPACA6 failed to induce the cultured cells to adhere to or bind with the plasma membrane of oocytes. Therefore, it is speculated that these proteins regulate fusion via an IZUMO1-independent pathway or act as mediators of fusion downstream [1].

### 3.6. SLLP1 (Sperm Lyzozyme-Like Acrosomal Protein) and Its Role in Egg–Sperm Fusion

SLLP1, or Sperm Lyzozyme-Like Acrosomal Protein 1, is a protein localized in the acrosome of sperms and is a specialized vesicle essential for fertilization. It belongs to the lysozyme-like protein family and has been implicated in the enzymatic breakdown of glycoproteins and glycolipids within the zona pellucida (ZP) of oocytes. The ZP is a critical barrier surrounding the egg, and its interaction with sperm during fertilization requires enzymatic activity to allow sperm entry. SLLP1 is thought to aid in the remodeling of the zona pellucida, facilitating sperm penetration by breaking down components such as glycosaminoglycans in the ZP matrix [66]. SLLP1 plays a crucial role in sperm–egg binding and fusion by enhancing sperm–oocyte adhesion through interactions with specific glycoproteins on the oocyte surface. Its function is important for species-specific fertilization, making it a potential target for understanding and addressing male infertility. Furthermore, SLLP1 could provide insights into contraceptive development by targeting the sperm–egg fusion process [66].

### 3.7. Other Accessory Proteins That Participate in the Fertilization Process

In the last decade, various studies have described some additional molecules that work in association with essential factors of the fusion process. Here, we also briefly describe some accessory proteins that help mediate the fertilization or fusion of gametes.

#### 3.7.1. Testis-Specific ADAMs and Their Associated Proteins Support the Fusion Process

Disintegrin and metalloproteinase (ADAM) protein family members are key regulators of various biological events, such as cell migration, cell adhesion, and cell interactions [67]. The testis-specific ADAM proteins ADAM1B and ADAM2 heterodimerize to form fertilin, which is localized to the sperm plasma membrane and works during the sperm fusion process [68]. *Adam2* KO mice were infertile; however, they were not involved in the egg and sperm fusion process [69]. On the other hand, *Adam1b* KO mice were completely fertile, although both ADAM2 and ADAM1B were absent from the sperm of *Adam1b* KO mice, which indicates that ADAM1B might play a supportive role in fertilization [70].

To date, more than 10 proteins have been reported that are involved in sperm migration and are required for ADAM3 localization in spermatozoa [71,72]. However, in this review, we have only discussed the roles of CMTM2A and CMTM2B in ADAM3 localization due to their overlapping functions in fertilization.

CMTMs are divided into eight family members from CMTM1–8 and are clustered on three different regions of human (3, 14, and 16) and mouse (8, 9, and 14) chromosomes [73,74,75]. Human CMTM1 has only a single homolog in mice, while human CMTM2 has two homologs in mice, Cmtm2a and Cmtm2b, which are specifically expressed in human and mouse testes [76]. Subsequently, Cmtm2a and Cmtm2b are evolutionarily conserved and share similar functions. Furthermore, localization experiments revealed that CMTM2A and CMTM2B are specifically localized on the sperm head [77]. Recent studies have demonstrated their functional importance in spermatogenesis by generating mutant Cmtm1, Cmtm2a, and Cmtm2b mice using CRISPR/Cas9 genome editing technology. Cmtm1 KO mice were completely fertile with no alterations in reproductive parameters. Subsequently, Cmtm2a and Cmtm2b double-mutant mice were generated, as both genes are homologs of the human CMTM2A gene, and both have higher levels of similarity at the genomic and proteomic levels. The double-mutant mice displayed normal development but produced no pups when mated with wild-type females. Further studies indicated that epididymal sperm have motility defects and have a reduced ability to bind to the zona pellucida. A more detailed investigation revealed that the mutant spermatozoa lack the sperm surface protein ADAM3 [77]. Interestingly, CMTM2B was also absent on the spermatozoa of Adam3 KO mice, while CMTM2A was still present. These findings indicated that CMTM2A and CMTM2B are essential for the localization of ADAM3 and male fertility in mice.

#### 3.7.2. Sperm-Borne Phospholipase C Zeta-1 Is Essential for the Induction of Ca^2+^ Changes During Fertilization

Sperm–oocyte fusion induces Ca^2+^ oscillation that triggers the resumption of the meiotic cell cycle and subsequent egg activation, blockage of polyspermy, and early embryonic development [78]. Several sperm-borne oocyte activation factor (SOAF) candidates have been proposed by microinjection studies [79]. Over the years, several experimental studies have suggested that phospholipase C zeta 1 (PLCZ1) fulfills all prerequisite criteria of the soluble sperm factor responsible for the generation of Ca^2+^ oscillations in mammalian fertilization. Furthermore, PLCZ1 acts as a physiological stimulus for the release of Ca^2+^ from the oocyte endoplasmic reticulum via the inositol 1,4,5-trisphosphate (InsP3) signaling pathway [80,81].

Numerous clinical studies have provided evidence in support of the direct link between defects or deficiencies in the PLCZ1 gene and male infertility [78,82]. However, a recent study based on Plcz1 KO mouse models developed using CRISPR/Cas9 technology reported that no defects in spermatogenesis, sperm motility, morphology, or acrosome reaction rates were observed in Plcz1 KO mice compared to a previous report. Plcz1 KO male mice can sire healthy offspring, albeit with significantly reduced pups per copulation (~25.8%). More than 50% of fertilized oocytes with Plcz1 KO sperm showed egg activation failure, abnormal activation or polyspermy, and a lower preimplantation developmental ability. Furthermore, the role of PLCZ1 in Ca^2+^ oscillation and oocyte activation was investigated using intracytoplasmic sperm injection (ICSI). Both Plcz1 KO spermatozoa and sperm heads, when used individually to perform ICSI, failed to induce intracellular Ca^2+^ changes, as well as oocyte activation and early embryo development [83]. However, at a high concentration of Plcz1 KO sperm, polyspermy increased up to ~80%. Interestingly, all the monospermic fertilized oocytes displayed atypical, delayed, but clear intracellular Ca^2+^ spikes, although these were lower in number and frequency. A consistent increase in the number of Ca^2+^ spikes was recorded with an increase in the number of fused Plcz1 KO spermatozoa. Additionally, fertilization of oocytes with Plcz1 KO spermatozoa resulted in a delay of both zona pellucida block to polyspermy (ZPBP) and plasma membrane block to polyspermy (PMBP). The delayed and low-frequency Ca^2+^ oscillation induced by PLCZ1 deficiency was consistent with the delayed and incomplete cortical reaction of ZPBP. Thus, PlCZ1 is critical for both ZPBP and PMBP to ensure monospermic fertilization and subsequent successful oocyte activation [83].

#### 3.7.3. Role of DC-STAMP Domain-Containing Protein 1/2 (DCST1 and DCST2) During Fertilization

DC-STAMP domain-containing protein 1 and 2 (DCST1 and DCST2) are critical transmembrane proteins expressed on the sperm membrane, and are essential for successful fertilization through their role in mediating sperm–egg fusion. These proteins are homologous to the dendritic cell–specific transmembrane protein (DC-STAMP), which is involved in membrane fusion in other cellular contexts. DCST1 and DCST2 are required for the structural and functional assembly of the fusion machinery during fertilization. Genetic studies in mice have shown that sperm lacking DCST1 or DCST2 can bind to the oocyte but fail to fuse with the egg membrane, resulting in fertilization failure despite normal motility and capacitation [84].

Mechanistically, DCST1 and DCST2 are believed to coordinate with other key fusion factors such as IZUMO1 and its oocyte receptor JUNO. They may function as molecular adaptors or stabilizers that help organize the fusogenic complex, promoting the necessary conformational changes in the membranes to enable lipid bilayer merging. Additionally, DCST1 and DCST2 are implicated in post-capacitation changes in sperm, potentially modulating membrane fluidity and protein distribution, which are prerequisites for fusion competency. Their precise molecular interactions remain under investigation, but their loss-of-function phenotypes underscore their indispensable role in ensuring the final stages of gamete fusion and successful fertilization [85].

## 4. Membrane Lipid Remodeling During Cell Fusion

Membrane lipid remodeling is a critical process during cell fusion, including sperm–egg fusion in fertilization. This remodeling involves dynamic changes in the composition and organization of lipids in the plasma membrane to facilitate membrane merging. Key events include the redistribution of phospholipids, cholesterol efflux, and the formation of microdomains enriched in fusogenic lipids, such as phosphatidylserine and phosphatidylethanolamine. These changes increase membrane fluidity and destabilize the bilayer, creating favorable conditions for membrane fusion. For instance, the sperm membrane undergoes capacitation-associated cholesterol efflux, mediated by albumin and lipoproteins in the female reproductive tract, which is essential for the acrosome reaction and subsequent membrane fusion. Similarly, in the oocyte, tetraspanin-enriched microdomains help organize key fusion proteins, such as IZUMO1 and JUNO, in lipid raft-like structures to optimize their interactions [86].

Fusogenic proteins often interact with specific lipids to induce curvature and promote fusion pore formation. For example, proteins such as syncytins in placental fusion and viral fusogens, such as influenza hemagglutinin, exploit lipid remodeling to mediate membrane deformation and fusion. The role of phospholipid scramblases and flippases in redistributing lipids across the bilayer is also critical in ensuring that membrane asymmetry is disrupted, a prerequisite for membrane merging. Aberrations in lipid remodeling can impair cell fusion, as seen in conditions such as infertility and placental dysfunction. Thus, understanding the molecular mechanisms of lipid remodeling offers insights into its critical role in facilitating membrane fusion and its potential as a therapeutic target in reproductive medicine [87].

## 5. Current Challenges and Future Directions

Despite the progress in understanding reproductive biology, the study of egg and sperm fusion at the molecular level faces a significant challenge due to the limited availability of human data. Research in this area is primarily constrained by the following factors.

### 5.1. Ethical, Data Scarcity, and Practical Limitations

Human gametes are not easily accessible for experimental purposes. Collecting human eggs and sperm for research is often difficult due to ethical concerns, especially in studies that require direct manipulation or observation of gamete fusion. Additionally, gametes are produced in relatively low numbers, making large-scale experiments challenging [88]. Human studies in this area are often restricted to clinical fertility settings, where data may be limited to certain populations (e.g., those seeking assisted reproductive technologies). This creates a gap in understanding the genetic diversity and molecular mechanisms of fusion in the general population, including those with unexplained infertility [89].

### 5.2. Model Organisms’ Specificity

Model organisms such as mice, zebrafish, and Drosophila have been instrumental in understanding gamete fusion, but they are not perfect representations of human biology. Several key differences exist and have been highlighted.

#### 5.2.1. Species-Specific Pathways

While mouse models have provided insight into sperm–egg fusion proteins, not all of these mechanisms are conserved in humans. Some gamete fusion proteins, such as Izumo1 and Juno, may have different homologs or interact with different sets of molecules in humans. This makes it difficult to extrapolate findings from mice directly to human fertility.

#### 5.2.2. Incomplete Representation of Human Fertility Issues

Many model organisms lack certain aspects of human fertility, such as the complex hormonal regulation or the wide variety of infertility causes seen in humans, such as genetic mutations in non-coding regions of the genome, epigenetic modifications, or environmental factors. In mouse models of infertility, disruptions to sperm–egg fusion proteins such as Izumo1 can lead to infertility, but these models may not capture the full complexity of human infertility disorders, especially when considering the genetic and environmental contributions that might influence gamete function in humans [90].

## 6. Future Directions

### 6.1. CRISPR-Cas9 for Functional Gene Studies

One of the most promising directions in fertility research is the use of CRISPR-Cas9 gene-editing technology. This approach has revolutionized how scientists study gene function by allowing precise, targeted modifications to the genome. It holds significant potential for addressing the limitations mentioned above [91].

CRISPR-Cas9 allows researchers to create precise knockouts or mutations in specific genes involved in egg and sperm fusion, enabling a clearer understanding of how each gene contributes to fertility. By knocking out genes such as Izumo1 or Juno in human models, researchers could simulate infertility conditions and study the exact mechanisms of fusion failure [92].

### 6.2. Humanized Model Systems

CRISPR-Cas9 can be used to generate humanized models of fusion, such as human pluripotent stem cells (hPSCs) that differentiate into sperm and egg-like cells. This would provide a better model to study human-specific genetic interactions in gamete fusion.

### 6.3. High-Resolution Imaging for Molecular-Level Understanding

In addition to CRISPR-Cas9, high-resolution imaging techniques will be crucial for understanding the molecular interactions that govern egg and sperm fusion. These techniques will allow researchers to observe these processes at a level of detail that was previously unattainable. Technologies such as super-resolution microscopy and fluorescent imaging can enable scientists to visualize real-time molecular interactions between the sperm and egg. This would provide insights into how fusion proteins move and interact during fertilization at a cellular level. Molecular dynamics including high-resolution imaging could allow researchers to visualize the conformational changes of fusion proteins such as Izumo1 and Juno during gamete fusion, providing a clearer understanding of how these proteins trigger the fusion process [93,94].

### 6.4. Integration of Omics Technologies

Another important direction for future research is the integration of multi-omics approaches (e.g., genomics, transcriptomics, proteomics, and epigenomics) to gain a deeper understanding of gamete fusion and fertility. Understanding the genetic mutations and expression profiles of genes involved in gamete fusion will help identify rare genetic variations that impact fertility. It can also reveal how gene expression patterns change during gametogenesis and fertilization. The role of epigenetic regulation in fertility is becoming increasingly apparent. Investigating how epigenetic modifications affect the expression of genes involved in fusion could provide new insights into infertility [93,94].

### 6.5. Advancing Fertility Treatments

By identifying key genes involved in gamete fusion and understanding how they work at the molecular level, we can develop targeted therapies for infertility. For instance, if a genetic mutation in Izumo1 is identified in a male patient, CRISPR-based therapies or gene therapy could be explored to restore fertility.

### 6.6. Personalized Medicine

As fertility research advances, integrating genomic data could allow for personalized fertility treatments. Patients with infertility could undergo genetic testing to identify specific fusion-related genetic defects, guiding the use of assisted reproductive technologies (ARTs), such as IVF or ICSI [95].

It is important to note that in assisted reproduction techniques such as ICSI, the natural signal transduction mechanisms leading to oocyte activation are bypassed. While calcium ionophores are sometimes used to artificially stimulate oocyte activation, these methods do not fully replicate the complex calcium signaling events that occur under physiological conditions, especially in humans. This difference may have implications for understanding and addressing challenges in human fertility, as the absence of physiological calcium signaling may impact embryonic development and overall success rates of assisted reproduction.

### 6.7. Broader Implications for Reproductive Health

Beyond infertility, understanding the molecular mechanisms of gamete fusion could help prevent reproductive health disorders, such as miscarriages, which often occur due to errors during fertilization. This knowledge can lead to new preventive strategies and improve assisted reproductive techniques.

## 7. Summary

ARTs are progressing on a daily basis. However, it has been reported that only 34% of the embryos that are created through IVF treatment are transferred back to the uterus, and 85% of transferred embryos fail to produce live births. Recently, studies have demonstrated that genes involved in fertilization events are important players in controlling the success of egg and sperm fusion reactions. Although both IZUMO and CD9 are considered mandatory to orchestrate sperm–egg interactions, integrins, disintegrins, and other receptors also facilitate sperm–oolemma adhesion during fertilization. Recently, an in vivo rescue approach discovered that SOF1, TMEM95, and SPACA6 are also essential for the interactions, and disruption of these genes leads to failure of the acrosome reaction during fertilization. In short, harnessing the mechanisms by which gene products mediate sperm–egg fusion will further enhance our knowledge and provide new insights into human-assisted fertilization.

Molecular insights into the roles of CD9 and CD81 in sperm–egg fusion have significant implications for assisted reproductive technologies (ARTs). The essential role of CD9 in organizing membrane domains and stabilizing the IZUMO1–JUNO complex highlights its potential as a biomarker for oocyte quality in in vitro fertilization (IVF) procedures. Defects in CD9 or its associated pathways could explain some cases of unexplained infertility, where sperm binding occurs without successful fusion. Similarly, understanding the compensatory role of CD81 may guide strategies to enhance oocyte receptivity by modulating membrane dynamics or fusion machinery. Advanced imaging and biochemical studies on these tetraspanins could inform the development of targeted interventions to improve fertilization rates, particularly in patients undergoing intracytoplasmic sperm injection (ICSI). Moreover, leveraging these insights could pave the way for novel contraceptives by disrupting critical protein interactions required for membrane fusion, offering dual benefits in fertility management [43].

## 8. Conclusions

The study of genes orchestrating egg and sperm fusion reactions has unveiled critical insights into the molecular mechanisms underlying human fertility. Advances in gene identification, functional analysis, and imaging technologies have significantly enhanced our understanding of how specific genetic factors contribute to successful fertilization. Despite these advancements, there remain substantial challenges related to limited human data, the specificity of animal models, and the complexity of gamete fusion processes.

Understanding the molecular mechanisms underlying egg and sperm fusion has far-reaching implications beyond gene editing therapies, particularly in the realm of diagnostics for unexplained infertility. Fusion mechanisms involve a complex interplay of proteins, lipids, and signaling pathways, such as those mediated by JUNO, IZUMO1, CD9, and integrins, which are essential for gamete recognition and membrane fusion. Aberrations in these pathways may underlie cases of idiopathic infertility, where no clear cause is identified through conventional diagnostic methods. Advances in studying these mechanisms have led to the identification of potential biomarkers, such as defective IZUMO1–JUNO interactions or abnormal lipid rafts in gamete membranes, which could serve as diagnostic indicators. High-resolution imaging, proteomics, and single-cell transcriptomics can now be utilized to detect subtle defects in these pathways, offering insights into unexplained infertility cases. Additionally, this understanding could pave the way for the development of precision-based interventions, such as tailored protein supplementation or membrane repair therapies, to overcome fusion-related fertility barriers. By integrating this knowledge into diagnostic workflows, clinicians could achieve more accurate diagnoses and personalized treatment strategies, offering new hope to couples facing infertility challenges.

Understanding the genes involved in egg and sperm fusion is critical for advancing fertility treatments. Key insights, such as the roles of Izumo1 (sperm) and Juno (egg), highlight the importance of specific fusion proteins in successful fertilization. Despite advancements, challenges remain due to limited human data and the species-specific nature of model organisms. Future research should focus on CRISPR-Cas9 gene editing and high-resolution imaging to investigate the genetic and molecular mechanisms of gamete fusion in humans. Clinical applications include personalized genetic screening for infertility, gene therapy for correcting fusion-related mutations, and enhanced ARTs, such as IVF and ICSI, tailored to individual genetic profiles. These advancements will improve diagnostic precision, provide targeted treatments, and offer new options for fertility preservation and non-invasive fertility.

## Figures and Tables

**Table 1:** IZUMO1 and its associated proteins function in Fertility.

Protein	Key Findings in Knockout Models	Fertility Outcome
IZUMO1	Zumo1-deficient male mice produced normal sperm and formed vaginal plugs, but sperm failed to fuse with eggs [27].	Males were sterile; direct injection of sperm into the egg cytoplasm restored fertility.
ACE3	Interacts with IZUMO1 in sperm but disappears post-acrosome reaction. Deficiency did not impair fertilization [28].	Ace3 deletion had no effect on fertility in vivo or in vitro.
JUNO	Juno-deficient female mice produced eggs that failed to fuse with wild-type sperm [30].	Females were infertile; JUNO is critical for binding and polyspermy prevention.
